# Expert collaboration for safe perinatal stabilization of neonates with select critical congenital heart defects at non-cardiac centers

**DOI:** 10.1186/s12887-026-06887-3

**Published:** 2026-04-23

**Authors:** Christian Brickmann, Kilian Ackermann, Marcus Krüger, Helge Mommsen, Katja Tschositsch, Christoph Scholz, Julia Hauer, Renate Oberhoffer-Fritz, Peter Ewert, Ann Sophie Kleinpaß

**Affiliations:** 1 Department of Pediatrics, School of Medicine and Health, Kinderklinik Muenchen Schwabing, Technical University of Munich, TUM University Hospital, Munich, Germany; 2 Clinic for Neonatology, Muenchen Klinik, Munich, Germany; 3 Clinic for Obstetrics and Gynecology, Muenchen Klinik, Munich, Germany; 4 Department of Pediatric Cardiology, School of Medicine and Health, Technical University of Munich, TUM University Hospital, Munich, Germany; 5 German Heart Center, TUM University Hospital, Munich, Germany

**Keywords:** Congenital Heart Disease, Perinatal Management, Paediatric Cardiology, Neonatal Outcomes, Interprofessional Collaboration

## Abstract

**Objective:**

To evaluate mortality and morbidity among neonates with select critical congenital heart defects (cCHD) delivered and initially managed at a Level-III perinatal center without in-house paediatric cardiac surgery, and to determine how many required immediate cardiac interventions versus stabilization prior to transfer.

**Design:**

Retrospective single-center cohort study (2018–2022) at a Level-III perinatal center with neonatologists and paediatric cardiologists on-site but no paediatric cardiac intervention unit. Inclusion of all neonates with prenatally or postnatally suspected cCHD delivered at the center and managed until transfer or discharge.

**Interventions:**

Structured neonatal care including prostaglandin therapy, respiratory support, and coordination with a tertiary cardiac center. Immediate non-surgical cardiac interventions were performed in-house when necessary.

**Main outcome measures:**

Pre- and postnatal diagnosis, neonatal mortality, morbidity, need for immediate cardiac intervention, postnatal bonding, and timing of transfer.

**Results:**

A total of 115 neonates were included; 94.0% had a prenatal cCHD diagnosis. Immediate non-surgical cardiac intervention was required in 3 neonates (2.6%) including emergency atrial septostomy performed on-site. The remaining 112 neonates (97.4%) were stabilized without the need for immediate intervention before transfer. Prostaglandin E1 infusion was initiated in 67.0% of neonates to maintain ductal patency. Median time to transfer was 4 days (IQR 3–7). Postnatal bonding occurred in 52.7% of cases despite the complexity of care. No neonates experienced acute in-house mortality during stabilization. Overall mortality was 9.6%, with 81.8% of deaths occurring after transfer and 18.2% following in-house palliative care based on prenatal counselling decisions.

**Conclusions:**

Most neonates with cCHD can be safely stabilized at a Level-III perinatal center prior to transfer without the need for immediate surgical cardiac intervention. The model allows for effective care and maternal bonding without increased acute mortality. These findings support collaborative perinatal management outside surgical cardiac centers for selected cases.

## Introduction

Congenital heart disease (CHD) is the most common congenital anomaly, with an estimated prevalence of 0.6–1.2% of all live births [1–6]. Among these, approximately 15–25% are classified as critical congenital heart defects (cCHD) with systemic low cardiac output, requiring intervention via cardiac catheterization or surgery within the first year of life therefore making it a significant contributor to neonatal morbidity and mortality [3, 7–12]. Despite advancements in diagnostic and surgical techniques, the mortality rate for neonates with cCHD remains substantial, with estimates ranging from 10% to 28% within the first year of life [7, 13–18].

Prenatal diagnosis of cCHD via fetal echocardiography plays a pivotal role in improving neonatal outcomes by enabling structured perinatal management [1, 11, 19, 20]. This multidisciplinary approach involves neonatologists, fetal cardiologists, paediatric cardiologists, cardiac surgeons, and obstetricians, ensuring a seamless transition from fetal to postnatal care. Existing guidelines classify CHD and the according immediate postnatal risk for morbidity into low-risk, minimal-risk, and high-risk categories, guiding perinatal management strategies [19, 21–23]. Low-risk defects, such as shunt lesions i.e. Ventricular (VSD) or Atrial Septum Defects (ASD), generally do not require immediate intervention and can be managed in outpatient settings. Minimal-risk defects, including ductus-dependent pulmonary or systemic perfusion, necessitate early stabilization with prostaglandin infusion before transfer to a paediatric cardiology center. Prenatally diagnosed High-risk defects, such as those requiring immediate postnatal non-surgical (e.g., balloon atrial septostomy/septoplasty) or direct surgical intervention (for example in patients with in Total Anomalous Pulmonary Venous Return (TAPVR) or Hypoplastic Left Heard Syndrome (HLHS) with Intact Atrial Septum), demand prompt coordination between neonatology and paediatric cardiology teams [19, 21–23].

The benefits of early postnatal bonding between mother and child are well established, particularly in neonates with preexisting medical conditions [24]. Bonding refers to both skin-to-skin contact between the mother and newborn and the early initiation of breastfeeding. It has been associated with improved short- and long-term neurodevelopmental and psychological outcomes. However, neonates requiring urgent postnatal intervention often face logistical and medical challenges that may delay or limit early maternal contact [25]. Additionally, the global shortage of highly trained paediatric cardiology specialists further complicates the immediate availability of expert care, increasing the need for well-structured interprofessional collaboration [25].

Although existing guidelines recommend delivery in or near a paediatric cardiology center [2], limited research has explored the feasibility and outcomes of neonates with cCHD delivered in perinatal centers without an on-site paediatric cardiology unit [1]. This may not be feasible in many regions due to geographic, infrastructural, or staffing limitations. Smaller or more remote perinatal centers often face logistical barriers, including limited access to tertiary facilities or delayed transfer capabilities. In such settings, having structured perinatal protocols and on-site neonatology teams, in collaboration with off-site paediatric cardiologists, may be essential for ensuring early stabilization and safe outcomes. Prior studies suggest that with adequate prenatal planning and structured postnatal management, neonates with cCHD can be safely stabilized in high-level perinatal centers before transfer to specialized cardiac centers [1, 10, 26, 27]. This approach optimizes resource allocation in tertiary cardiology units, ensuring that hospital beds remain available for neonates requiring definitive interventions rather than prolonged pre- or post-surgical care.

This study examines the outcomes of neonates with cCHD delivered and initially managed in a Level-III perinatal center without an in-house paediatric cardiology unit but paediatric cardiologists in staff.

## Methods

### Study design and objectives

This study is a retrospective single-center cohort analysis. The primary objective was to evaluate mortality and morbidity outcomes in neonates with cCHD who were delivered and initially managed outside a dedicated paediatric cardiology center without Paediatric Cardiac Surgery. The secondary objective was to perform a descriptive analysis of cCHD subtypes and associated clinical parameters to assess the feasibility of structured perinatal care in this setting, contributing to the ongoing discussion on optimizing perinatal management for neonates with cCHD.

### Study centers

The Primary (Perinatal) Cardiac Center (PCC) is located at the Perinatology Center of Munich Klinik Harlaching (MüK). It comprises a Neonatology and Obstetrics Department providing comprehensive prenatal care and is designated as a Level-III Perinatology Center. The PCC annually manages approximately 50 very low birth weight preterm infants (< 32 weeks gestational age and < 1500 g), as well as 20–25 neonates diagnosed with critical congenital heart defects (cCHD). Senior pediatric cardiologists are part of the in-house staff, and a pediatric cardiologist is available on-call outside regular working hours, ensuring presence within 30 min when needed. Catheter-based interventions are available 24/7.

The PCC operates in close collaboration with the German Heart Center Munich (DHZ), a university-affiliated pediatric cardiology center. The DHZ—also referred to as the Secondary (Specialized) Cardiac Center (SCC)—does not have an in-house obstetric unit or maternity ward. It specializes in pediatric cardiology including cardiac surgery and provides ECMO therapy, which is not available at the PCC. DHZ is one of few centers globally to perform invasive procedures outside their own main cardiac center. Specialized neonatal transport for cCHD patients is available 24/7 and is coordinated by the DHZ. The SCC serves as a dedicated pediatric cardiology center, without obstetric or perinatal services [28]. There are no cost differences or economic benefits associated with treatment at either the PCC or the SCC. Both centers operate under the same public healthcare framework and are equally accessible to patients regardless of insurance or socioeconomic status.

### Study population

The study cohort included all neonates diagnosed prenatally or postnatally with cCHD who were delivered at MüK between January 1, 2018, and December 31, 2022. Neonates were included if they received perinatal and neonatal management at this center until transfer to a tertiary paediatric cardiology facility for intervention, surgery, or discharge. Discharge could have been after primary care directly from PCC, after specific care from SCC or after readmission at PCC. Exclusion criteria comprised neonates who received prenatal care at the study center but were delivered elsewhere.

Clinical data were obtained retrospectively from electronic patient records in the hospital’s Clinic Information System platform and medical reports from the German Heart Center Munich. Data collection was conducted by the study investigators, ensuring standardized documentation.

### Study endpoints

#### Primary endpoints

- Mortality and morbidity rates, classified as follows:
  - Neonates receiving primary palliative therapy
  - Neonates who died before transfer to a paediatric cardiology center
  - Neonates who died after transfer or following surgical or interventional procedures

#### Secondary endpoints

- Functional classification of CHD subtypes:
  - Ebstein Anomaly
  - Left Outflow Obstruction
  - Transposition of Great Arteries (TGA)
  - Left to Right-Shunt
  - Heterotaxy-Syndrome
  - Univentricular Defects
  - Tetralogy of Fallot (ToF)
  - Arrythmia
  - Others
- Clinical parameters, including:
  - Prenatal care and timing of first cardiologic evaluation
  - Mode of delivery
  - Gestational age, birth weight, length, and head circumference
  - Postnatal bonding and initial admission location: Neonatal Intensive Care Unit (NICU), Intermediate Care Unit (IMC) or Maternity ward. Bonding refers to both skin-to-skin contact between mother and infant as well as the early initiation of breastfeeding. For the purpose of this study, bonding is defined as uninterrupted maternal–infant contact lasting at least 60 min immediately after birth
  - Need for in-house neonatal interventions (e.g., Rashkind atrial septostomy, prostaglandin infusion)
  - Duration of stay before transfer to a tertiary cardiac center
  - Rates of retransfer to the perinatal center after cardiac intervention
  - Neonatal thriving (weight and head circumference changes from birth to discharge incl. Percentiles)
  - Final discharge status

### Statistical analysis

Descriptive statistical analyses were performed to summarize patient characteristics and clinical outcomes. Continuous variables were reported as Median with Interquartile Range, while categorical variables were expressed as absolute numbers and percentages. The Wilcoxon-Mann-Whitney test was used for non-parametric comparisons, with a significance level set at *p* < 0.05. Fisher’s exact test was applied for categorical contingency analyses. Statistical analysis was conducted using GraphPad Prism version 10 and “SPSS 27”.

## Results

### Study cohort and demographics

A total of *n* = 115 of *n* = 118 neonates with cCHD were included in this study. Demographic characteristics are summarized in Table 1, detailing gestational age at prenatal cardiologic evaluation and birth, as well as birth weight, length, head circumference, and mode of delivery. The median gestational age at birth was 39 + 1 weeks, with a median birth weight of 3090 g (min. 1100 g / max. 4390 g), *n* = 19 (16,5%) babies were pretem < 37 + 0 weeks of GA and *n* = 5 (4,3%) babies were less than < 34 + 0 weeks of GA. Neonates were delivered via vaginal delivery in *n* = 48 (41,7%) of cases, while *n* = 53 (46,1%) were delivered via cesarean section, including *n* = 4 (3.5%) emergency procedures or in *n* = 10 (8,7%) of cases using a vacuum extraction. 3 children were excluded due to palliative care according to Trisomy 18 (2x) and Hydrops fetalis with multiple organ dysplasia (1x).

**Table 1:** Comparison of the demographic pregnancy features and postnatal adaptation

Demographic data				
	*n* = 115	(%)	Median	IQR 25/75%
GA at birth (week + day)	Min. 31 + 5	Max. 41 + 4	39 + 1	37 + 6 / 40 + 1
GA at 1. cardiologic prenatal Consultation (week + day)	Min. 13 + 5	Max. 40 + 2	31 + 4	28 + 3 / 34 + 2
Sex (male / female)	58 / 57	50,4 / 49,6		
Singleton / Gemini	103 / 12	89,6 / 10,4		
Birth Weight (g)			3090	2605 / 3465
Birth Length (cm)			50	47 / 52,5
Birth Head circumference (cm)			34	32,5 / 35
APGAR 1			9	7 / 9
APGAR 5			9	8 / 10
APGAR 10			10	9 / 10
Umbilical pH			7.29	7,21 / 7,33
Base excess			-3.6	-6.4 / -1.5
*Delivery Mode*:				
Spontanous. vaginally	48	41,7		
Ceasarean Section	53	46,1		
Emerg. Cesarean section	4	3,5		
Vacuum extraction	10	8,7		

*Abr*.: *GA* Gestational Age, *IQR* Interquartile range; *g* grams, *cm* centimetre

### Pre- and postnatal diagnosis and functional classification of cCHD

**Fig. 1:**
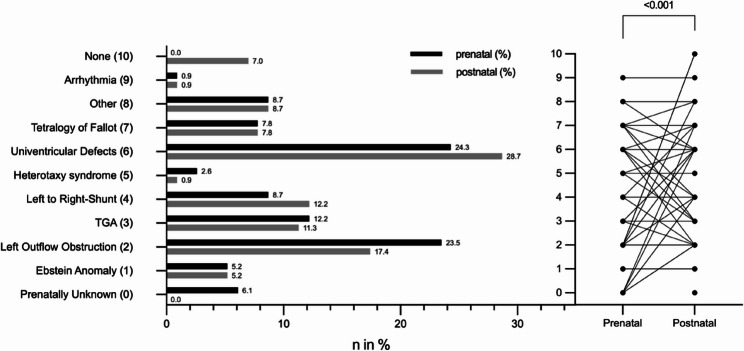
Comparison of pre- and postnatal diagnosis. On the left side are total numbers of the prenatal cCHD groups in black and the postnatal diagnosis in grey lines. The single points on the “Prenatal – Postnatal” comparison (right side) are matching the diagnosis on the left. The connecting lines show corresponding diagnosis from first prenatal and postnatal examination. Abr.: TGA = Transposition of the Great Arteries

Prenatal detection of cCHD was achieved in *n* = 108 (93.9%) of cases, with *n* = 101 (87.8%) of neonates receiving prenatal care at the perinatal center. Postnatal confirmation of the prenatal diagnosis was significantly different (*p* < 0.01; OR 2.2) and only possible in *n* = 79 (72.2%) of 108 cases (Table 2). cCHD was classified into nine major diagnostic groups (Fig. 1), with the most common being:

**Table 2:** Comparison of pre- and postnatal diagnosis. Description of necessary postnatal intervention and the kind of postnatal care, outcome parameters after referral to a specific cardiac center

Diagnosis				
	*n* = 115	(%)	*p*-value	OR (95%CI)
Prenatal Care at perinatal center	101	87,8		
Prenatally suspected cCHD	108	93,9		
Prenatal diagnosis confirmed	78	72,2	< 0.01	2.2 (1.4–3.5)
*Postnatal Care*				
	n=	(%)	Median	IQR
PGE	77/115	67		
Intervention inhouse	3/115	2,6		
Postnatal Bonding	59/115	52,7		
*Postnatal ward*:				
NICU	100/115	87		
IMC	6/115	5,2		
Maternity Ward	9/115	7,8		
Duration until Transfer/Discharge (days)	94/101		4	3 / 7
Time at home until Readmission (days)	5/101		115	25.5 / 156.5
*Outcome*				
	n=	(%)	Median	IQR
Duration at SCC (days)			14	8 / 28
*Readmission Ward*				
PICU	16/45	36		
IMC	24/45	53		
Paediatrics	5/45	11		
Duration to final Discharge (days)			19	7 / 33
Final Discharge PCC / SCC	72 / 26	73 / 27		
*Mortality*				
Deaths in total	11	9,5		
Acute PCC	0	0		
Palliative PCC	2	18,2		
External (SCC/at home)	9	81,8		

*Abr*.: *OR* Odds ratio; *cCHD* critical Congenital Heart Defect, *PGE* Prostaglandin E, *NICU* Neonatal Intensiv Care Unit, *IMC* Intermediate Care, *PICU* Paediatric Intensiv Care Unit, *IQR* Interquartile range, *PCC* Primary (Perinatal) Cardiac Center, *SCC* Secondary (Specialized) Cardiac Center

- Univentricular Defects: *n* = 28 (24.1%) (prenatal), *n* = 33 (28.7%) (postnatal)
- Systemic Outflow Tract Obstruction: *n* = 27 (23.5% (prenatal), *n* = 20 (17.4%) (postnatal)
- TGA: *n* = 14 (12.2%) (prenatal), *n* = 13 (11.3%) (postnatal)
- Left-to-Right Shunt Lesions: *n* = 14 (8.7%) (prenatal), *n* = 14 (12.2%) (postnatal)
- ToF: *n* = 9 (7.8%) (prenatal), *n* = 9 (7.8%) (postnatal)

Further detected diagnoses included Ebstein Anomaly *n* = 6 (5.2%), Heterotaxy Syndrome *n* = 3 (2.6%), Arrhythmias *n* = 1 (0.9%), and other defects *n* = 10 (8.7%), including Truncus Arteriosus Communis (TAC) *n* = 3 (2.6%), Double Aortic Arch *n* = 3 (2.6%), Right Aortic Arch *n* = 1 (0.9%), Pulmonary Valve Stenosis *n* = 1 (0.9%), Bilateral Superior Vena Cava *n* = 1 (0.9%), Cardiac Tumors (Rhabdomyoma) *n* = 1 (0.9%), and Atrial Aneurysm with compression of the right ventricle *n* = 1 (0.9%). Pre- and postnatal diagnosis did not differ in these patients. Evaluation of Risk Stratification Assessment resulted in *n* = 25 (23,3%) Low Risk Cases, *n* = 62 (58%) Medium Risk Cases and *n* = 20 (18,7%) High Risk Cases.

The prenatally suspected diagnosis of children with no postnatal CHD confirmation were in *n* = 8 (7.0%) cases were all suspected Aortic Coarctation (AoC). cCHD diagnosis (*n* = 7; 6.1%) without prenatal detection were: postnatally confirmed AoC in *n* = 3 (2.6%) cases; Left to Right Shunt in *n* = 2 (1.7%) cases; Univentricular Heart defect *n* = 1 (0.9%), Tetralogy of Fallot *n* = 1 (0.9%). The Heterotaxy case presented as complex cCHD with situs inversus abdominalis, right atrial isomerism, univentricular Heart Defect, Pulmonary Atresia and Malposition of the aorta.

### Postnatal management and initial care

Postnatal bonding was achieved in *n* = 59 (52.7%) of cases, with *n* = 9 (7.8%) of neonates initially admitted to the maternity ward. The majority of neonates required specialized care, with *n* = 100 (87.0%) admitted to the NICU and *n* = 6 (5.2%) to the IMC (Table 2).

Prostaglandin therapy was initiated in *n* = 77 (67.0%) of cases to maintain ductal patency. Cardiac interventions were performed in-house in *n* = 3 (2.6%) of neonates, emergency Rashkind atrial septostomy procedures performed by the SCC Team. All 3 cases were prentally unknown TGA with Restrictive Atrial Septum.

### Transfers and long-term outcomes

**Fig. 2:**
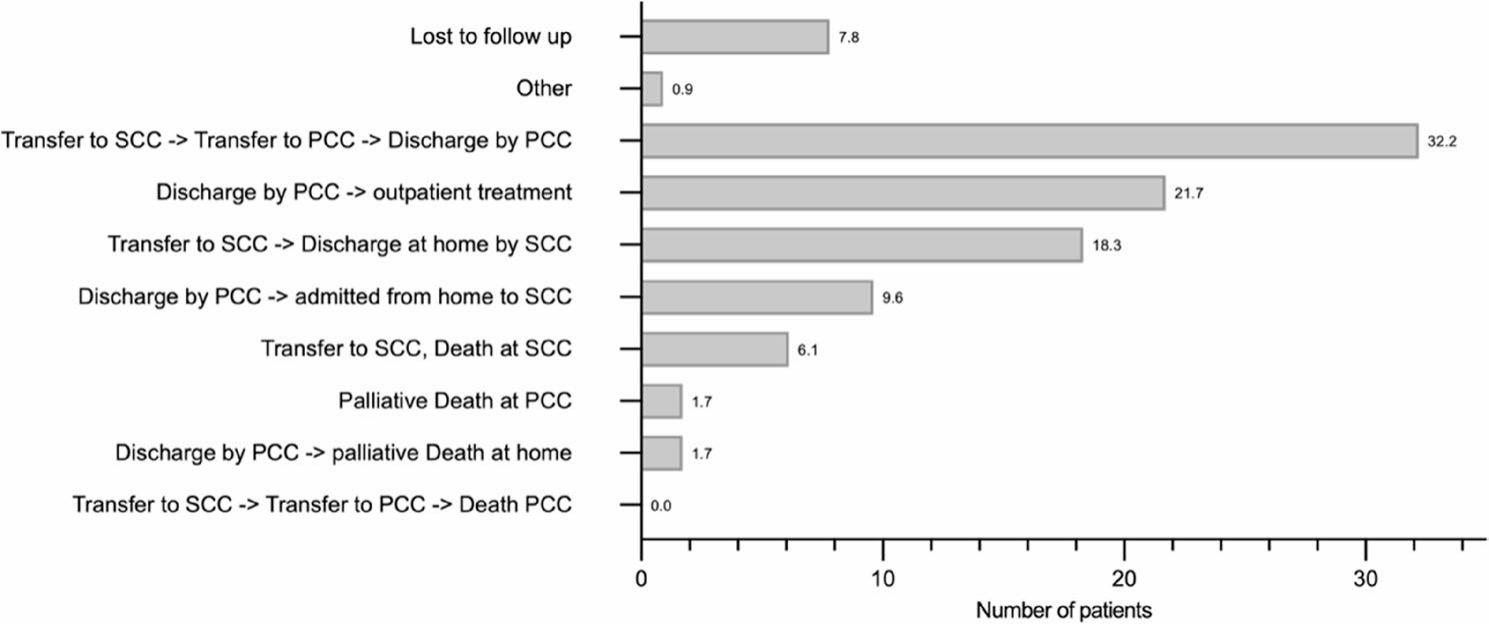
Patient Flow Diagram indicating patient referral routes after birth. The single “Other” transfer included treatment at a Otorhinolaryngology department prior to SCC treatment. Abr.: SCC = German Heart Center Munich Secundary (Specialized) Cardiologic Center); PCC = Muenchen Klinik (Primary (Perinatal) Cardiologic Center)

Some neonates were being discharged directly home following postnatal stabilization and cardiologic assessment with a median time of outpatient surveillance of 115 days before Admission at SCC. Neonates requiring intermediate surgical or interventional treatment had a median duration of hospitalization before transfer to SCC of 4 days. Among them, 45 neonates (39.1%) were later retransferred from SCC to PCC after cardiac intervention for continued non-surgical post-operative care (i.e. feeding issues, antibiotic treatment, anticoagulation therapy, decongestive therapy) (Fig. 2).

**Fig. 3:**
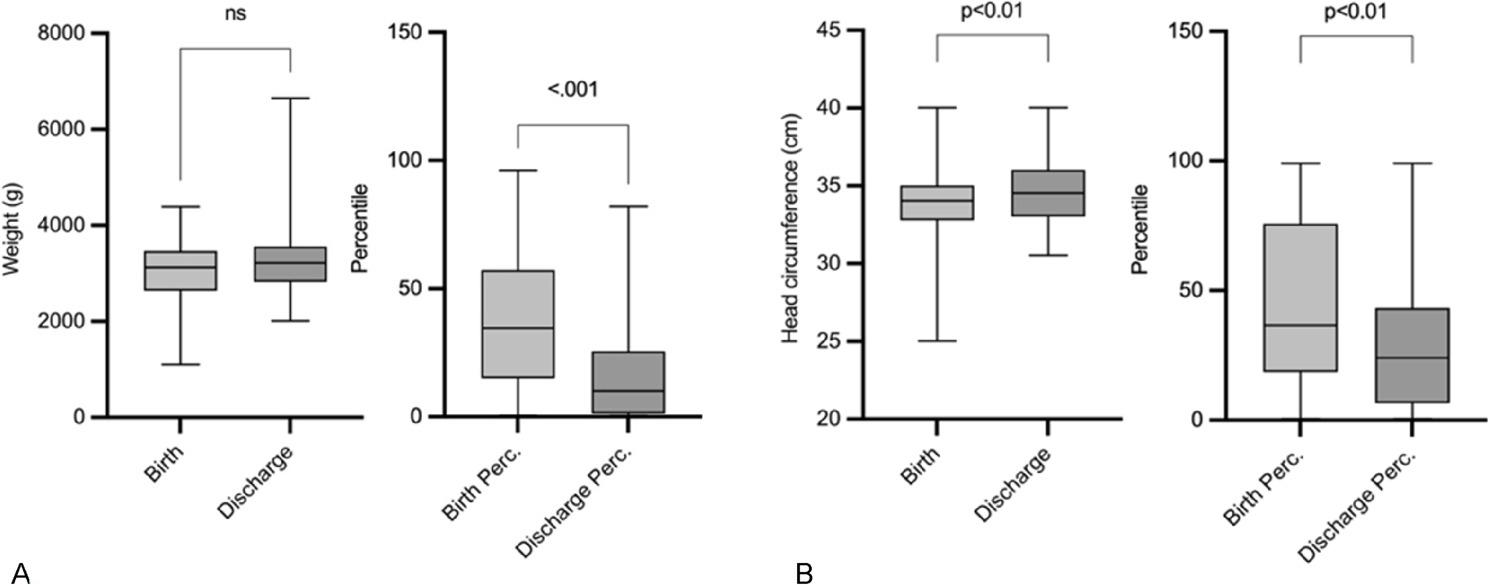
Evaluation of neonatal thriving by comparison of Bodyweight (**A**) and Head Circumference (**B**) after birth and at final discharge in total and in Percentiles. Abr.: ns = not significant.; g = gram; cm = centimetre; Perc. = Percentile

The median duration of hospitalization at SCC was 14 days, with neonates remaining at the perinatal center for an additional 19 days post-retransfer before final discharge (Table 2). Assessment of neonatal growth parameters from birth to discharge revealed a significant increase in head circumference (*p* < 0.05), while weight gain remained statistically non-significant. The assessment of the corresponding percentiles however showed a significant decrease (Fig. 3).

### Mortality and morbidity

The overall mortality rate in this cohort was *n* = 11 (9.6%), consistent with previous studies on CHD outcomes in HIC (Table 2). Among the deceased neonates:

- *n* = 2 (18.2%) died palliatively in-house following a prenatal decision for non-interventional management.
- *N* = 9 (81.8%) died externally following transfer to the paediatric cardiology center.
- No neonates experienced acute in-house mortality during initial postnatal stabilization.

## Discussion

Studies focusing on neonates with CHD born outside specialized paediatric cardiology centers are limited. Our study contributes to this underexplored area by evaluating the outcomes of neonates with cCHD managed in a Level-III perinatal center equipped with a paediatric cardiology focus, in close collaboration with a university paediatric cardiology and cardiac surgery center, concentrating on the short-term outcome. An indicator for the severity of cCHD evaluated in our cohort is the amount of prostaglandin dependent defects, even when taking into account, that these did not include diagnoses in which urgent surgical or catheter intervention were anticipated.

We did observe a significant difference between the pre- and postnatal echocardiographic diagnosis with 23 different Types of Mismatches between pre- and postnatal diagnosis. However, this finding is not based on inexperienced prenatal obstetric observation but for one on the fact, that prenatally suspected aortic coarctation was not clinically apparent after postnatal assessment in 8 cases: an often-occurring issue in fetal echocardiography. 7 cases have not been seen in the initial echocardiography (therefore resulting in a change in diagnostic classification after birth) and another 8 cases were mismatches between an initial diagnosis of ToF or Univentricular Defects. These shifts in pre- and postnatal diagnosis reflect the evolving nature of certain lesions during gestation; even minor changes in anatomical features such as ventricular size over time can result in reclassification of the diagnosis after birth without having an impact on the clinical severity of postnatal treatment.

But despite the difference of initial prenatal and final postnatal diagnosis our results align with previous studies showing that prenatal diagnosis through fetal echocardiography, followed by specialized perinatal management, substantially improves postnatal outcomes. In our cohort, 94% of neonates were diagnosed prenatally, allowing for optimal planning and coordination between neonatologists, fetal cardiologists, paediatric cardiologists, and obstetricians [6, 15, 22]. This early and correct detection ensured timely and adequate management, contributing to the favourable outcomes observed in this cohort.

Notably, the 10.3% overall mortality rate in our study mirrors the findings of Anagnostou and is significantly lower than the reported 25% mortality rate for neonates with cCHD in other studies [1]. Additionally, no neonatal deaths that were acutely or non-palliative occurred in-house, which is an important finding, demonstrating the effectiveness of neonatal stabilization protocols (especially when urgent interventions are not anticipated) and the absence of delayed interventions. These findings confirm that neonates with cCHD can be successfully managed in a structured perinatal care setting without an increased risk of acute cardiac-related mortality.

Our study emphasizes the importance of postnatal bonding, which has been well-documented as a critical factor in improving neonatal outcomes especially for infants with significant medical burdens. Immediate postnatal bonding was facilitated in 53% of neonates in our cohort which is a notable achievement given the complex needs of this population. Despite the technical and staffing demands involved in managing neonates with cCHD, our model allowed for the immediate postnatal bonding of mothers and infants, thereby enhancing their short- and long-term outcomes. These findings corroborate the existing literature on the benefits of early mother-infant contact, particularly in high-risk newborns [24]. Newborns with cCHD have an increased caloric need for growth. Long-term intensive-care medicine also takes its toll on nutritional input and therefore musculoskeletal thriving, but brain growth is an important factor for adequate neurocognitive development. Despite total increase in measurements of weight and head circumference concerning the total values the according percentiles showed a significant decrease from birth to final discharge. This underlines the impact of the morbidity o cCHD and is well in accordance with published data.

Regarding management pathways, our study supports the feasibility of delivering and managing neonates with minimal-risk CHD in a Level-III perinatal center, with timely transfer to a paediatric cardiology center for necessary interventions. Neonates with ductus-dependent pulmonary or systemic perfusion were transferred after an average of 4 days, which is comparable to the timelines reported by other studies. The transfer system we employed—ensuring that paediatric cardiology beds were utilized for necessary surgical or interventional procedures rather than for prolonged pre- or post-procedural care—proved efficient and effective [2].

For neonates with high-risk CHD requiring postnatal care but no immediate surgical interventions, our study indicates that initial stabilization by neonatologists, followed by timely intervention by paediatric cardiologists, is a safe and effective approach. Only 2.6% of neonates required in-house intervention, and these were managed without any significant increase in mortality or morbidity. Three Rashkind-Manouevers due to TAC with restrictive atrial septum that were prenatally unknown were executed at the PCC ward postnatally by staff from the SCC. There were no other emergent surgery procedures. All children could be stabilized and then transported to the SCC. The SCC specializes in catheter procedures under sonographic control in an outpatient setting at external collaborating centers. This approach, where cardiology specialists arrived shortly after birth or were available prenatally, ensured that these infants received the necessary care without compromising immediate stabilization [6–8, 10]. The teams are aware, that the lacking of surgical backup options in case of complications poses a risk. This setting profits from short transportation duration within the city. When calculating the time necessary for postnatal stabilization and diagnostic procedures this would suffice to prepare the OR no matter if inhouse or in a different building accessible only by transportation. The additional time for the transport itself would only marginally delay surgical intervention. Even conditions like TAPVR would be able to be stabilized and surgically treated in a timely manner. But we would like to emphasize, that we still consider this as only an emergency procedure and prefer timely prenatal transfer.

One of the key strengths of our study is the demonstration of no increase in morbidity or mortality despite the delivery of select high-risk neonates outside of a paediatric cardiology center. This affirms the viability of collaborative care models, particularly in regions with limited access to highly specialized centers. Our findings align with similar studies that have shown favourable outcomes when there is a well-coordinated approach between perinatal and paediatric cardiology teams, even when taking our specific setting into account. We are aware, that our model and settings in a high care region of a High-Income Country (HIC) positively influence the impact on mortality. None the less, the collaborative nature of our care model ensured that select cardiac interventions were performed at the optimal time, which contributed to the low mortality rate and the absence of acute postnatal deaths within the perinatal center [5, 20, 21].

Our study has several limitations. First, as a retrospective single-center analysis, the findings may not be fully generalizable to other institutions with different infrastructures and care models. Another limitation is the lack of long-term follow-up data, including neurodevelopmental and quality-of-life outcomes, which are essential for assessing the broader impact of this care model. Our cohort was assembled pre-, peri- and post COVID-19 era. COVID restrictions in Germany influenced the medical sector, including logistics, staff availability, and infection control practices. However, obstetric and neonatology units experienced relatively limited disruptions in our setting. Separation of mother and newborn occurred only in cases where the mother was severely symptomatic or critically ill with COVID-19. In our cohort, no neonates were separated solely due to maternal SARS-CoV-2 positivity.

## Conclusion

We could demonstrate that select neonates with cCHD can be effectively managed in a Level-III perinatal centre with a paediatric cardiology focus until an appropriate time for intervention or surgery is reached. Importantly, no neonates died acutely in-house, underscoring the effectiveness of structured neonatal stabilization and early intervention strategies. Immediate postnatal bonding, an essential component of neonatal well-being could regularly be provided, facilitated by close collaboration between neonatologists and paediatric cardiologists. This model of cooperative care ensures efficient use of specialized cardiac beds while providing high-quality neonatal management closer to the families.

The findings suggest that a structured perinatal care approach, integrating fetal cardiology, neonatology, paediatric cardiology, and obstetrics, enables safe delivery and stabilization of neonates with select cCHD outside specialized paediatric cardiology centres. Given the increasing demand for specialized neonatal cardiac care and the global shortage of highly trained personnel, interprofessional collaborations between perinatal and tertiary cardiac centres offer a sustainable model for maintaining high-quality outcomes without increasing neonatal mortality. Future studies should explore long-term neurodevelopmental and quality-of-life outcomes in this patient population to further optimize care pathways.

## Data Availability

Data could be obtained by contacting the corresponding author.
